# Supplementary figures and images for: Lathosterol Oxidase (Sterol C-5 Desaturase) Deletion Confers Resistance to Amphotericin B and Sensitivity to Acidic Stress in Leishmania major
Source: mSphere. 2020 Jul 1;5(4):e00380-20. doi: 10.1128/mSphere.00380-20 (PMC7333571; doi:10.1128/mSphere.00380-20)

**WT**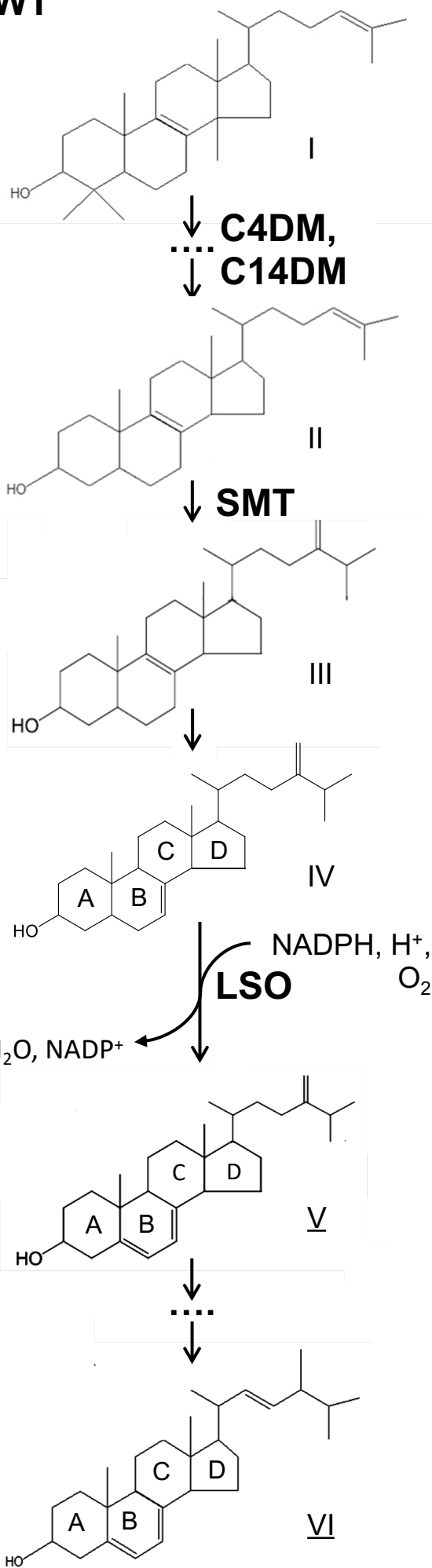***Lso*<sup>-</sup>**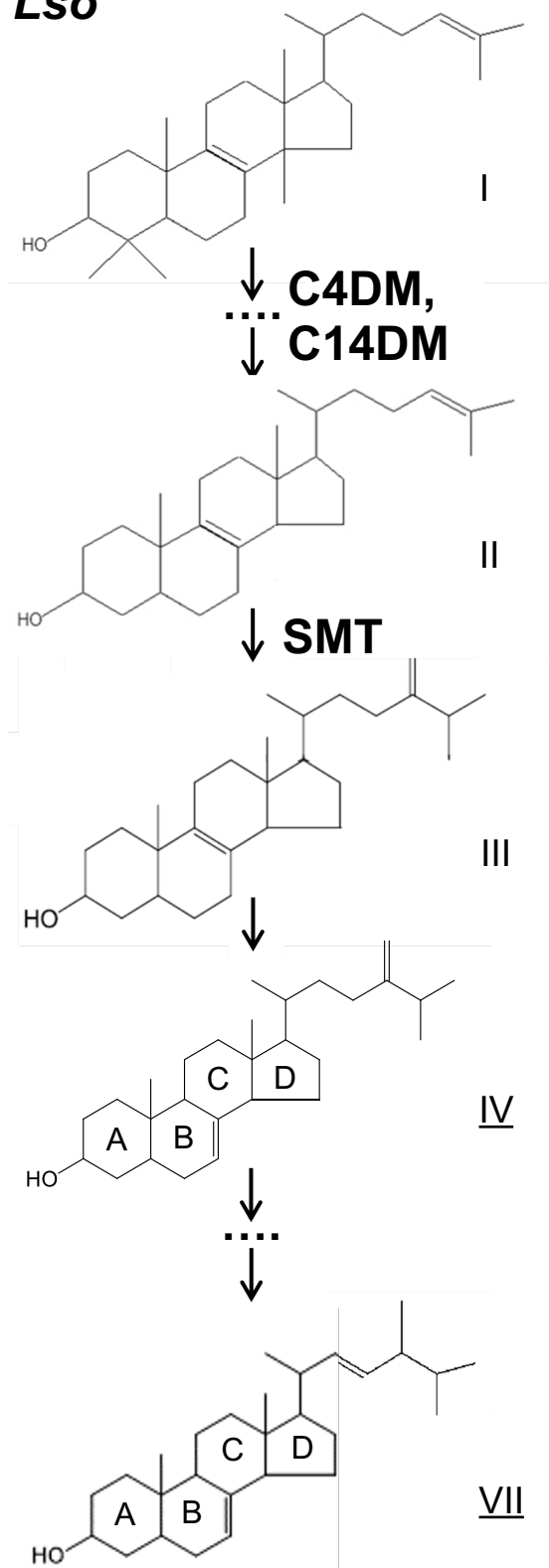

Supplement: FIG S1 [file mSphere.00380-20-sf001.pdf]

**A**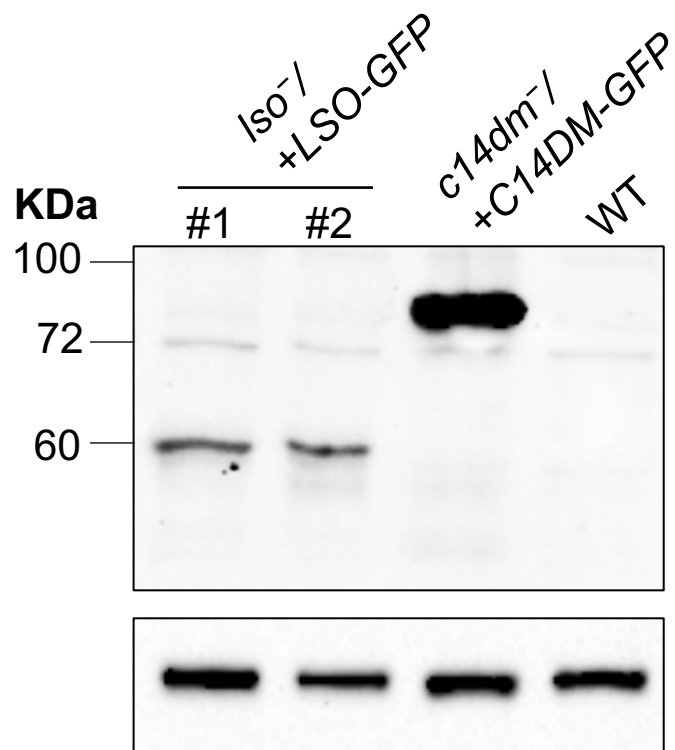**B**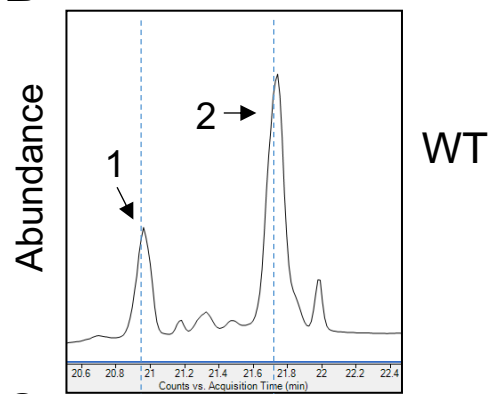**C**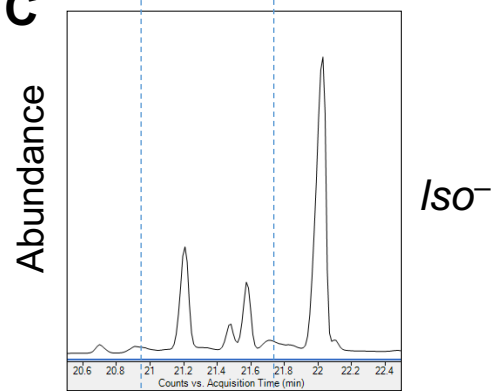**D**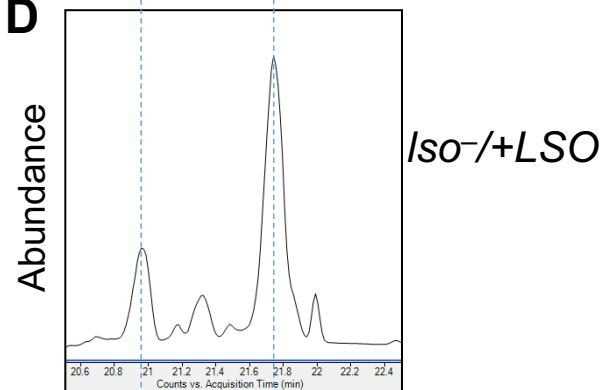**E**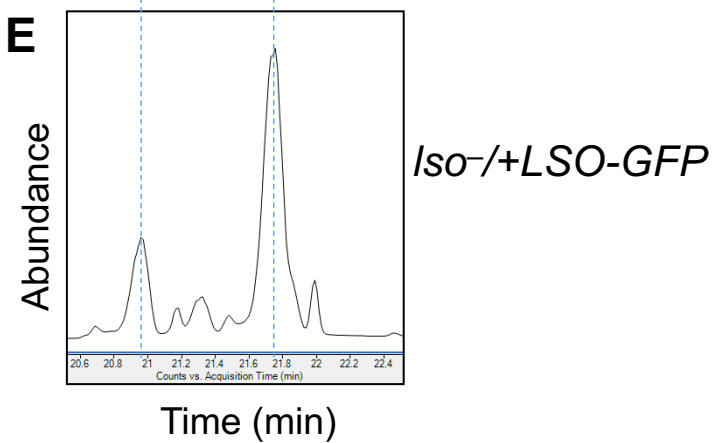

Supplement: FIG S2 [file mSphere.00380-20-sf002.pdf]

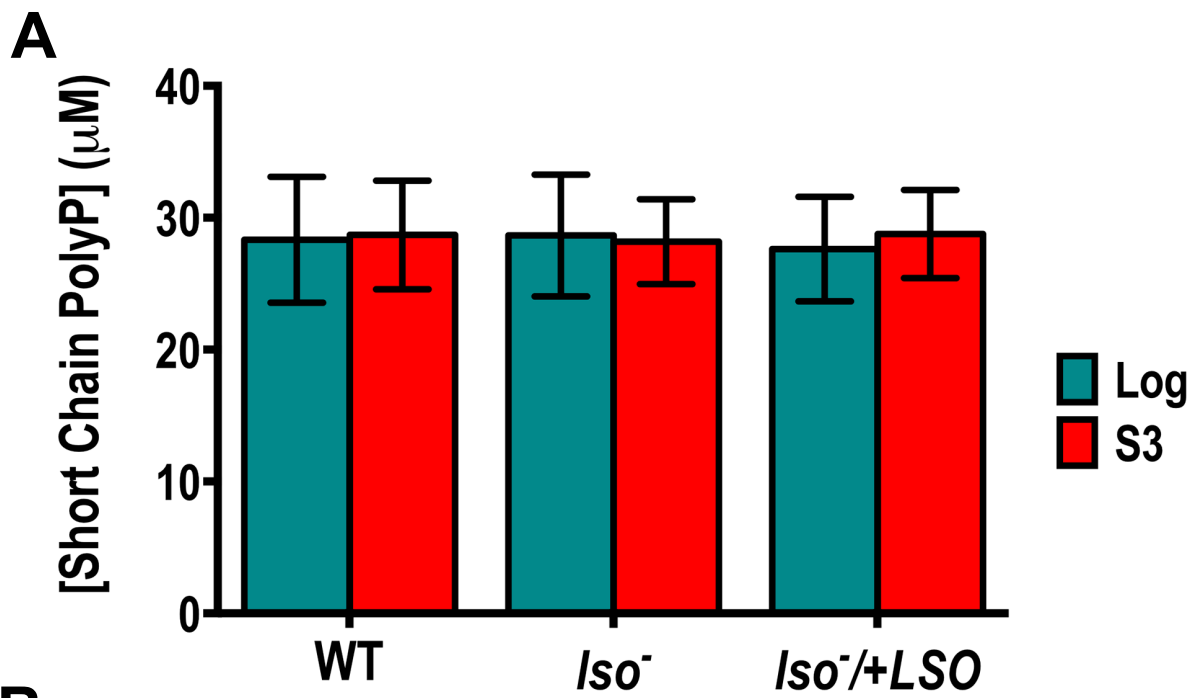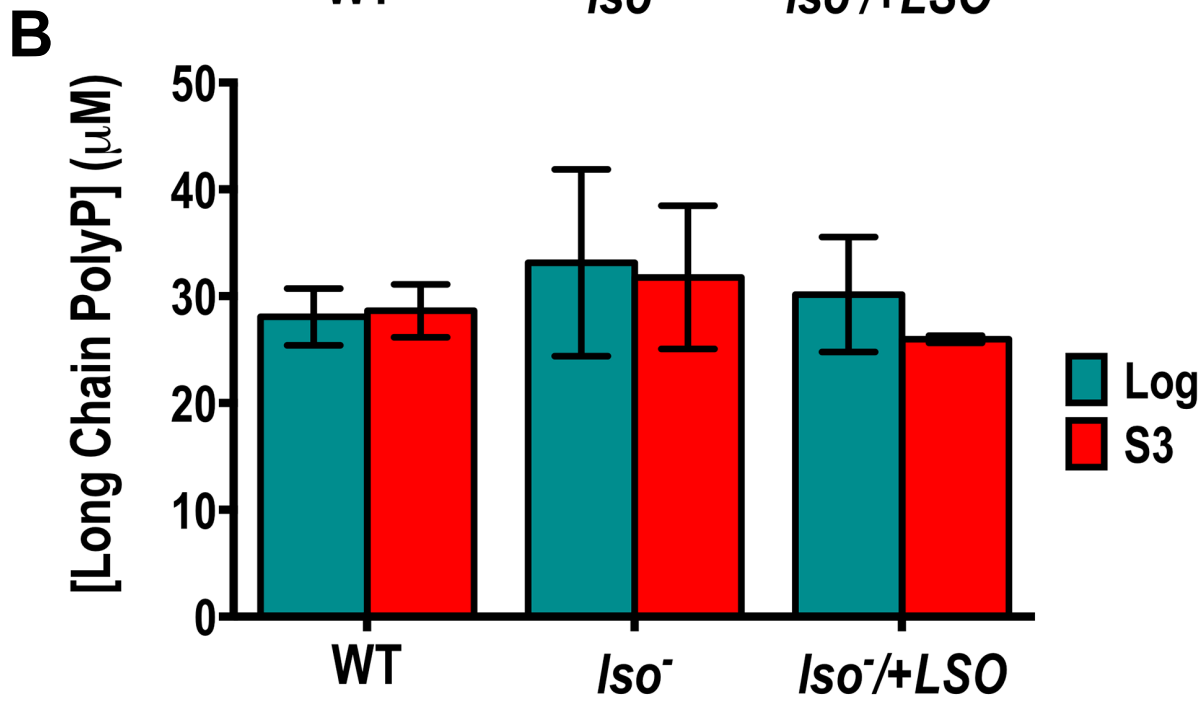

Supplement: FIG S3 [file mSphere.00380-20-sf003.pdf]

**A**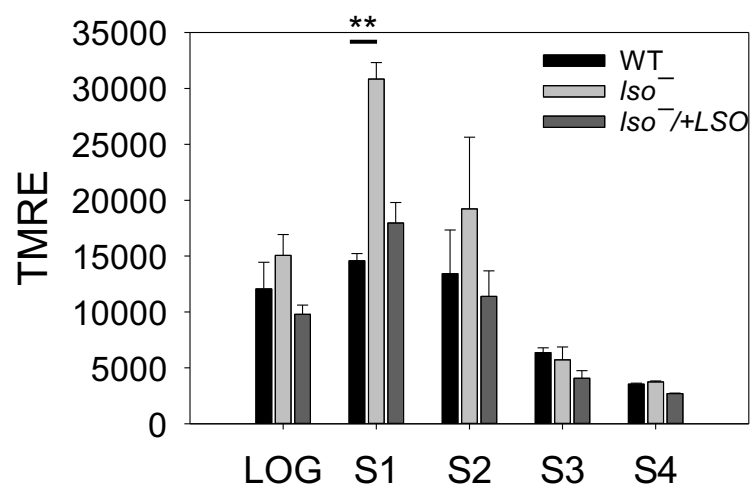**B**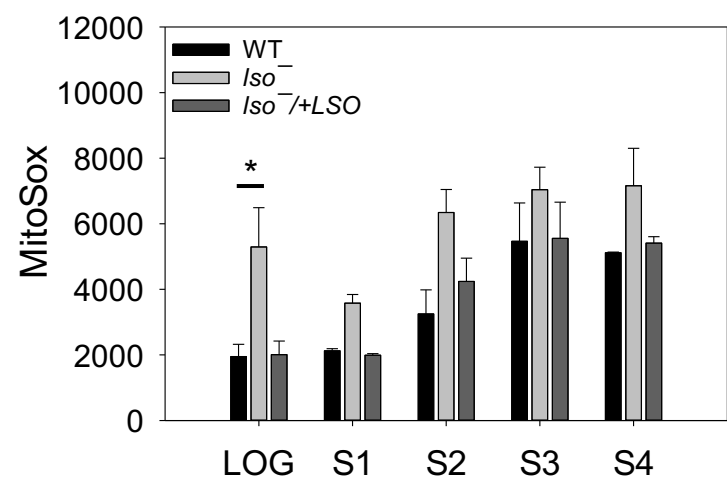**C**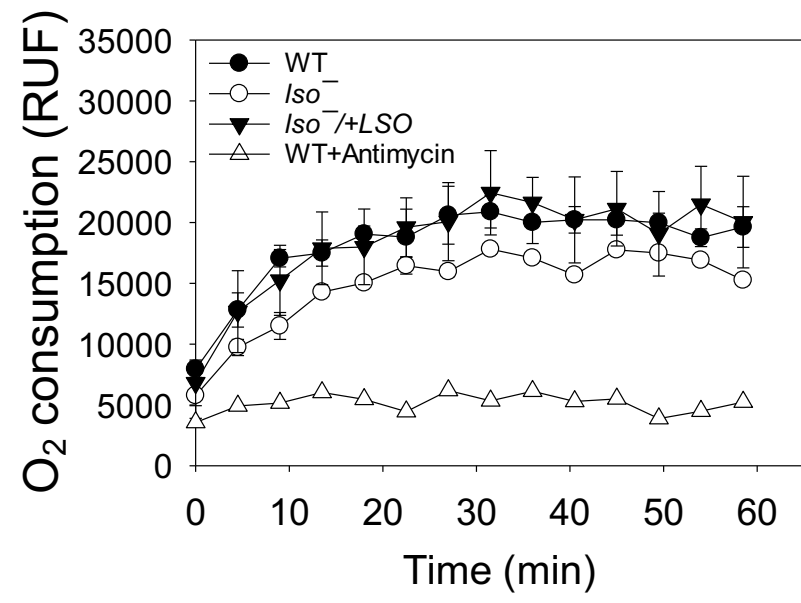

Supplement: FIG S4 [file mSphere.00380-20-sf004.pdf]
