# Supplementary material for: Lathosterol Oxidase (Sterol C-5 Desaturase) Deletion Confers Resistance to Amphotericin B and Sensitivity to Acidic Stress in Leishmania major
Source: mSphere. 2020 Jul 1;5(4):e00380-20. doi: 10.1128/mSphere.00380-20 (PMC7333571; doi:10.1128/mSphere.00380-20)
Supplement: TABLE S1 [file mSphere.00380-20-st001.pdf]

Table S1. List of oligonucleotides used in this study.

Primer #	Name	Purpose	Sequence
645	LSO-5'UTR-F-EcoRI	To amplify the 5'-flanking sequence	CATGATgaattcCACTGTACGTCGGCTCGTG
646	LSO-5'UTR-R-SpeI	To amplify the 5'-flanking sequence	GTCGCTactagtGCTTTCGAATGAGCCGGTG
647	LSO-3'UTR-F-SpeI-BglII	To amplify the 3'-flanking sequence	CGGACGactagtGGCTAGagatctGAGAAGGATCGGTCACCTTATTG
659	LSO-3'UTR-rev (HindIII)	To amplify the 3'-flanking sequence	GGCAGCaagcttCCTACGTACTTACTCGCAC
649	LSO-ORF-F-BamHI	To amplify the *LSO* ORF	TCAGTAggatccACCATGGACTTCGCCTTTCGCC
650	LSO-ORF-R-BamHI	To amplify the *LSO* ORF	GCACTggatccTTATGCGCCGACCTTCTTTGAGC
651	LSO-GFP-R-EcoRV	To generate the *LSO-GFP* fusion	GATGCTgatatcTGCGCCGACCTTCTTTGAGC
653	LSO-Probe 1-F	To generate the LSO probe for Southern blot	GAGTAACCGAGTCGGTTGTC
654	LSO-Probe 1-R	To generate the LSO probe for Southern blot	GTGAGAAGTGTCTCGACG
655	LSO-Probe 2-F	To generate the LSO probe for Southern blot	CTCTTCGCCTCCATCTCCTAC
660	LSO-Probe 2-R	To generate the LSO probe for Southern blot	GATCGCCAGAAGTTCACCAC
848	SCGs-F	For qRT-PCR analysis of SCGs	GAGGAGAACAATGTGCCAG
849	SCGs-R	For qRT-PCR analysis of SCGs	CCGCAGTGCCTCTGAGAG
709	28S rRNA-F	For qRT-PCR analysis of 28S rRNA	AAGATGGACCGGCCTCTAGT
710	28S rRNA-R	For qRT-PCR analysis of 28S rRNA	ATCCTTCCCCGCTCCAGTAT
